# Hemostatic Challenges in Pediatric Critical Care Medicine—Hemostatic Balance in VAD

**DOI:** 10.3389/fped.2021.625632

**Published:** 2021-02-26

**Authors:** Muhammad Bakr Ghbeis, Christina J. Vander Pluym, Ravi Ram Thiagarajan

**Affiliations:** ^1^Division of Cardiac Critical Care, Department of Cardiology, Boston Children's Hospital, Harvard Medical School, Boston, MA, United States; ^2^Division of Advanced Cardiac Therapies, Department of Cardiology, Boston Children's Hospital, Harvard Medical School, Boston, MA, United States

**Keywords:** hemostasis, ventricular assistance device, anticoagulation, thrombosis, bleeding, heparin, bivalirudin, TEG-PM

## Abstract

Ventricular assist devices (VAD) are used more in children. Safe and effective anticoagulation is required for successful management of children supported with ventricular assist devices. Developmental hemostasis, device hemocompatibility, plastic to body ratio, surgical variable techniques, lack of knowledge on pharmacokinetics of anticoagulants, and wide variability in anticoagulation protocols have all contributed to increased incidence of bleeding and thromboembolic complications. New collaborative learning networks, such as the ACTION network, provide opportunities to define best practices, optimize, and reduce anticoagulation related adverse events. ACTION was established Dec 2017. It consists of expert clinicians in heart failure, as well as researchers, parents, and patients, with goals to improve outcomes, share data, improve education and standard practice for children with heart failure (^1^, n.d). Changes in pediatric VAD anticoagulation strategy from using mainly heparin to DTI such as bivalirudin have helped reduce bleeding and clotting complications.

## Introduction

Since FDA approval of the Belin Heart EXCOR ventricular assist device (VAD) in North America over a decade ago (1) pediatric VAD use has increased with favorable reduction (>50%) in waiting list mortality and improved survival following heart transplantation (2). The 4th Annual Pediatric Interagency Report for Mechanical Circulatory Support (Pedimacs) published in 2019 reports that 1,031 devices were placed in 856 children at 44 centers during September 2012 through December 2019 (3). Increase in VAD use is largely due to increasing use of intracorporeal continuous flow devices (4). Despite increasing VAD use, hemocompatibility related adverse event, including bleeding and thrombosis with current antithrombosis agents remain among the significant challenges in children supported on VAD (5). Safety and efficacy of antithrombotic agents in these patients are in ongoing need of evaluation. While the use of antithrombotic therapies worldwide was consistent, variability in practices among VAD centers was noted (6). Achieving optimal anticoagulation and antithrombosis in VAD patients requires a balanced control of thrombin and platelets inhibition against physiologic hemostasis. In infants and young children, achieving this balance has been challenging due to several unique physiologic factors including developmental hemostasis as originally described by Monagle et al. (7). This report summarizes these challenges and describes the current antithrombotics use in children supported with VAD.

### Pediatric VAD Device Options

a. Berlin Heart EXCOR, a pulsatile paracorporeal pump, is the first and only Food and Drug Administration durable VAD for use in children as a bridge to transplantation. The Berlin Heart device is used as a single or biventricular assist. Placement requires median sternotomy and use of cardiopulmonary bypass. Berlin Heart EXCOR ventricular assist device can be used to support children weighing >3 kg up to adulthood, in the form of univentricular and biventricular support. With previous heparin based anticoagulation regimen (Edmonton Anticoagulation and Antiplatelet Protocol), the rate of stroke was consistently ~ 30%, but has since decreased over the last 5 years with the adoption of multiple interventions, including use of steroids to mitigate inflammation, (8) use of consistent team to manage anticoagulation (9), and adoption of DTI for primary anticoagulation (10).
b. Paracorporeal continuous flow devices include temporary circulation support devices such as Pedimag®/Centrimag®, Rotaflow®, and the Revolution® pumps (11). These are placed *via* median sternotomy and use of cardiopulmonary bypass is limited to supporting the most challenging patients whose size and anatomy necessitate use of this paracorporeal pump. Despite the described continuous flow technology increased risk of thrombosis in children with body surface area (BSA) <1.0 m^2^ (12), it's applications continue to evolve with tendency toward longer term support in select populations (13). TandemHeart is also a paracorporeal continuous flow device which is used more in the adult populations. One main advantage of this device is that cannulas are placed percutaneously so no need exists for thoracotomy, however the size of the cannulas is the limiting factor and thus pediatric experience remains limited (14).
c. Intracorporeal VADs are currently the most common VAD type placed in older children, composing around 50% as per the most recent annual Pediatric Interagency Registry for Mechanical Circulatory Support (Pedimacs) report. This shift in the use of VAD for children is attributed to advancements in device design with associated lower adverse event profile (3). Per Morales et al., 34% of Intracorporeal patients are still supported with the device at 6 months and nearly 20% at 1 year, which is in stark contrast to paracorporeal device types, which have close to zero patients remaining on device support at 1 year. They also estimated 50% of the patients are receiving Intracorporeal VAD as a bridge to candidacy (3). The HeartWare_ HVAD system (Medtronic, Mounds View, MN) is one of the earlier intracorporeal continuous flow pump to be used in larger children and adolescents even before FDA approval for adults only in 2012. The smallest known patient with this device is 2.7 years old and 13.1 Kg. It is reported that close to 50% of children with HeartWare devices are discharged home (15). The HeartMate VAD represents the newest in centrifugal continuous-flow VAD technology. Its optimized hemocompatibility profile, particularly HM3, and shear stress reduction makes it with the least side effects profile among all the devices. The smallest reported patient supported with this device is 8.8 years and 19.1 Kg. Per O'Connor et al., adverse events related to the HM3 device were uncommon so far (median 78 days, 2–646), with no episodes of pump thrombosis, pump dysfunction requiring operative exchange, or stroke (16). The Jarvik 2015 VAD is the most recent and promising intracorporeal continuous flow pump for infants and smaller children. The preliminary enrolment weight range is 8–20 kg with BSA range of 0.4–1.0 m^2^. As described in its website, www.pumpkintrial.com, the PumpKIN trial is a prospective, multi-center, single-arm feasibility study that will evaluate the safety of the Jarvik 2015 as a bridge to transplant or recovery in children. Of note, patients eligible for the PumpKIN trial are limited severe heart failure with two-ventricles (17).
  It is worthy to note that pediatric patients on intracorporeal devices are generally less ill, older, and are less likely to have congenital heart disease compared with patients on paracorporeal pulsatile or paracorporeal centrifugal flow devices (3).
d. Impella is a percutaneous ventricular assist device that is a micro-axial pump. Impella has recently been utilized more in pediatrics for short-term circulatory support. The most common indication of its implant was cardiogenic shock with variable underlying pathophysiology including cardiac allograft rejection, myocarditis, or cardiomyopathy (18, 19). It is inserted and secured percutaneously (or through a chimney graft) into the axillary or femoral artery, and then positioned in the LV across the aortic valve. The continuous blood flow that is pumped can vary based on the Impella generation, but most recently up to 5.5 l/min.

### Hemostasis and Age

#### Coagulation Cascade

Maturation related differences in the coagulation cascade in infants and children presents unique physiologic challenges compared to adults (7, 20). Clinicians are faced with the need to explore age related changes with each component of the coagulation cascade, both in physiologic or pathophysiologic states. These normal physiological differences in maturation of the coagulation cascade are referred to as “developmental hemostasis.” During fetal life, maternal coagulation factors are unable to penetrate the placental barrier. Coagulation factors synthesis in the fetus starts as early as 5.5 weeks (21). After birth, coagulation factors have different postnatal patterns of maturation toward adult values for most components by 6 months of age (22, 23). Some factors such as fibrinogen, Factors V, VIII, von Willebrand (vWF), and XIII are similar to mature adults even at birth, while Kuhle et al. reported levels of vWF and high-molecular weight multimers as well as vWF collagen binding activity remain increased during the first 2 months of life and then gradually decrease to adult levels (21). While at birth, plasma concentrations of the direct inhibitors AT (antithrombin) and HCII (heparin cofactor II) are ~50% of adult values. Then at about 6 months of age, plasma concentrations of AT and HCII reach adult levels, α2-M levels are nearly twice those of adult values and remain increased throughout childhood (21). It is however also worth noting that variations in AT activity are not fully explained by age and other factors may play a role (24, 25).

#### Platelets

The only available evidence for altered platelet function in children is in neonates and in *in vitro* studies. Michelson and colleagues reported neonatal platelets to be hyporeactive to the platelet-activating agents such as thrombin, adenosine, phosphate/ epinephrine, and thromboxane A2. Yet, the bleeding time in neonates, a reflection of platelet function, is normal due to increased red blood cell size, hematocrit, and vWF multimers (26).

### Hemostasis and Congenital Heart Defects

Congenital heart disease (CHD) is known risk factor for intravascular thrombosis due to altered flow dynamics that can predispose to areas of flow stasis, and/or higher shear stress, with platelet activation, and placement of thrombogenic artificial material. Patients who undergo cardiac surgery are at risk of both thrombosis and bleeding. These risks are higher in the <1 year age group (27). Additionally, delay in age based normalization of coagulation factors in children with CHD has also been documented. In children with CHD, maturation in coagulation factors occurs after 48 months of age (28). Given the body and vessels size for young children, there is an increased chance for chest re-exploration and post-operative bleeding following heart surgery. Increased bleeding may be secondary to an acquired dilutional coagulopathy in these patients with the use of large priming solution compared with the blood volume during cardiopulmonary bypass, as well as delayed maturation of coagulation factors (28). Other clinical factors associated with coagulation system abnormalities in patients with congenital heart disease may be related to the physiological effects of heart failure, which may include decreased hepatic coagulation factor production (25). Finally, other unique coagulation factor abnormalities in patients with single ventricle congenital heart disease is the reported increase in factor VIII following Fontan operation with increased thromboembolic risk (29).

These issues illustrate challenges to providing antithrombotic therapies to children supported with VAD. Careful consideration of developmental hemostasis is required in constructing safe and efficient antithrombosis protocols in children, particularly children with congenital heart disease.

## Thrombotic Complications and VAD

Thrombotic complications are among the most common serious adverse events in VAD patients, despite different attempts to protocolize antiplatelet and anticoagulant therapies with different ways to accurately monitor its effects (5, 30). Different reasons are believed to cause thrombosis in VAD patients that mainly involve contact mechanism from artificial surfaces of the device as well as high shear stress, both of which activate vascular endothelial cells as well as platelets, and white cells. Inflammation which has been observed in VAD patients is also believed to contribute to the prothrombotic state in these patients (31). Other proposed factors include turbulence at anastomosis sites, and heat generated by the device.

In the recent Pedimac registry report, the incidence of hemorrhagic stroke was noted to be 11%. This was lower compared to prior times and did not vary by device type (3). In the prospective trial of the Berlin Heart pediatric VAD device, stroke occurred in 29% (1). Similar to paracorporeal pulsatile flow devices, paracorporeal continuous flow devices, such as the Rotaflow (Maquet, Wayne, NJ) and the Centrimag/Pedimag (Abbott, Abbott Park, IL), were found to have a high rate of neurologic events at 24% (13). In pediatric HeartWare HVAD patients, neurologic events were reported as 15.8% of all causes of death. When compared to young adults, children had more early bleeding and more early and late neurologic dysfunction.

The recent increased use of DTI for paracorporeal VAD patients has further reduced the incidence of stroke on DTI therapy for all devices types to as low as 1.7 events per 1,000 days of support. The Stanford Modified Antithrombotic Guideline that utilized triple antiplatelet agents in addition to enoxaparin was shown to reduce the incidence of stroke in children supported with Berlin Heart EXCOR devices to 0.8 events per 1,000 days of support utilizing.

In terms of time to first neurologic event and ischemic stroke, intracorporeal continuous flow devices performed better than both types of paracorporeal devices, pulsatile and continuous flow. The incidence of stroke was not different for paracorporeal pulsatile or continuous flow devices (3). In the early HeartMate 3 VAD experience of pediatric and patients with congenital heart disease, stroke or pump thrombosis were not detected for median of 78 days of follow-up.

In comparison to children supported with VAD, the incidence of stroke in adults supported with intracorporeal devices is reported to be around 17% (32). Similar to children, HeartMate 3 (HM3) VAD, had a lower reported stroke than other devices with an incidence of 0.46 events per patient-year in the 1st month and down to 0.04 events per patient-year in the 2 year period (33).

## Pump Thrombosis

VAD pump thrombosis represents an increased risk for morbidity and mortality given association with device malfunction, thromboembolic events, and hemolysis. Factors related to pump thrombosis can be related to patient, device, or anticoagulation management. In adult patients, Bartoli et al. reported LVAD thrombosis in 2–13% patients with a continuous-flow LVAD, 4–13% with axial-flow devices, and 2% with centrifugal-flow devices (34). The incidence of pump thrombosis reported in adult patients on LVAD continuous flow devices ranged from 0.014 to 0.03 events per patient-year (35), The HeartMate III device had no reported pump thrombosis for the first 6 months in a large prospective trial (36), and in another retrospective large study (37) though there are case reports of pump thrombosis at 1 year (38).

In Pediatric VADs, device thrombosis occurs in 18% of pediatric patients with a paracorporeal pulsatile device (39). Pump thrombosis event rate on DTI was 3.7 per 1,000 patient-days for the Berlin Heart EXCOR VAD, and 13.7 per 1,000 patient-days of centrifugal-flow VAD support (11). Pump thrombosis in HeartWare device in children was reported as 11% in a multicenter study using the Pediatric Interagency Registry for Mechanical Circulatory Support (Pedimacs) registry (16). In the early experience of HM3 in the pediatric and young adult population, there was no reported pump thrombosis (16).

Overall, time to first device malfunction/thrombus in children was significantly better in intracorporeal devices types, than paracorporeal device types, with the paracorporeal pulsatile pumps performing significantly better than paracorporeal continuous flow devices (3).

Pump thrombosis can start as either fibrin-rich (red) or platelet-rich (white) thrombi. Fibrin thrombi form around stagnant blood flow whereas platelets thrombi form in areas of turbulence. Management of pump thrombosis included surgical or pharmacological. Some pharmacological options are intravenous thrombotic agents such as fibrinolytics (e.g., alteplase) and glycoprotein IIb/IIIa (GPIIb/IIIa) inhibitors (e.g., eptifibatide) (40). Most reports of tPA use for pump thrombosis is inconclusive with evidence of increased bleeding complications (35). Similarly, GPIIb/IIIa inhibitor also increased risk of bleeding complications (40). A common practice in adult VAD institutions for pump thrombosis includes intensifying antiplatelet therapies, increasing the International Normalized Ratio (INR) goal when warfarin therapy is resumed, and/or DTI targeting a high activated partial thromboplastin time (aPTT) (40). With increasing reports of HIT among VAD adult patients, some institutions now use Bivalirudin as the agent of choice for pump thrombosis (40). Other studies have shown that bivalirudin therapy for management of pump thrombosis was associated with high recurrence rates and suggest need for other therapies including surgical pump replacement (41). Similarly others found that surgical intervention resulted in less mortality, stroke, and resolution of hemolysis than a medical strategy alone (42).

Pediatric VAD pump thrombosis treatment is based on adults' experience with case reports of using bivalirudin and low-dose systemic tissue plasminogen activator (TPA). When there are concerns of hemolysis impact on kidney function or if the patient is close to a previous surgery, device exchange should be considered (43). ACTION is currently working on settings shared guidelines for pediatric VAD management including pump thrombosis protocols (44).

### Bleeding

Bleeding is similarly among the most common complications for VAD patients. Some known factors are the historic lack of a standardized approach to anti-coagulation and anti-platelet dosing or monitoring in children, surgical techniques due to size and complexity, as well as developmental hemostasis in children.

In adults, major bleeding events are the most common adverse event within the first 3–12 months of continuous flow LVAD implantation. Gastrointestinal hemorrhage was the most common site of bleeding (up to 40%, particularly after 3 months) (45, 46). Based on the Pediatric Interagency Registry for Mechanical Circulatory Support (PediMACS) report for outcomes of children supported with temporary devices, the most frequent early adverse events were bleeding (28%) (13). In the Australian retrospective report for children on HeartWare HVAD and Berlin Heart EXCOR VADs, major bleeding occurred in 39% of the patients, with the majority of these events happening while on unfractionated heparin and on more than one antiplatelet agent (47). In a meta-analysis for children on durable Ventricular Assist Devices (87% of which were Berlin Heart), the incidence of bleeding overall was 37%. Reported bleeding events included gastrointestinal bleeding 15%, intracranial hemorrhage 16% or chest re-exploration 23% (6). In children and young adults with HeartWare devices, the event rate of early bleeding was 2-times higher in children than in young adults, but the overall bleeding incidence was the same, 23% (16). In the early HeartMate 3 VAD experience of pediatric and patients with congenital heart disease, significant post-operative bleeding was uncommon and was reported in only two patients (6%) with a median age of 15.7 years (8.8–47.3) (16). These findings may help understand bleeding risk better given the fact that these are larger devices in smaller size patients.

The role of improved understanding of anticoagulation and antithrombosis management for pediatric VAD patients was more recently noted. In patients on Berlin EXCOR VAD, using the Sanford protocol, bleeding events incidence rate was 8.6 per 1,000 days of support (48). VanderPluym et al. reported in the largest multicenter experience of DTI use for anticoagulation therapy in pediatric paracorporeal VAD support, major bleeding present in 16% of the cases (2.6 events per 1,000 patient days of support on DTI) (10).

Additionally, bleeding events are minimized by following certain management tips. Achieving prompt hemostasis following the VAD placement is of importance, and was a fundamental requirement for patients in the DTI therapy protocol for example with 90% of patients starting DTI 12 h postoperatively or after (10). Cognizant anticoagulation management and monitoring in the early post-operative periods, as well as during switching between agents is highly recommended. Other management tips for bleeding while on VAD are by decreasing intensity of the antithrombotic regimen and/or discontinuing the antiplatelet agents.

### Anticoagulation Management

Hemostasis in pediatric patients on VAD is a common challenge, and the optimal antithrombotic therapy for children with VADs is unknown. Pediatric VAD anticoagulation and antithrombotic management is widely variable with a shift toward more aggressive antithrombotic therapy. This shift could be in part due to previous experience of high rates of thrombotic complications in children with paracorporeal pulsatile-flow devices. Furthermore, developmental hemostasis in children, and coagulation challenges brought on by the presence of CHD, a population at risk for needing VAD, continues to challenge clinicians managing VAD in the pediatric populations (27).

The three main known pediatric anticoagulation pediatric VAD protocols based on the historic timeline are illustrated below Table 1:

a. Edmonton Protocol: includes initiating unfractionated heparin when bleeding is minimal at 24–48 h post-implantation with long-term anticoagulation using Enoxaparin for patients ≤ 12 and warfarin for patients >12 months of age. Dual antiplatelet therapy starts at 48 h with dipyridamole begun at 4 mg/kg/day, followed by Aspirin 1 mg/kg/day divided twice daily after chest tubes removal. Thromboelastography with Platelet Mapping (TEG-PM) is used for both anticoagulation and antiplatelet titration.
b. Stanford Protocol: includes similar anticoagulation strategy starting with heparin and converting to Enoxaparin or Warfarin, but adds a 3rd anti-platelet agent and targets a weight based dose (Aspirin 30 mg/ kg/day, Dipyridamole 15 mg/kg/day, Clopidogrel 1 mg/kg/day), with no titration to effect based on TEG-PM, and recommends using use of prednisone for signs of inflammation (fibrinogen > 600 mg/dL) (48).
c. ACTION Direct Thrombin Inhibitor (DTI) Harmonization Protocol: Includes starting bivalirudin once surgical and coagulopathic bleeding has resolved with evidence of normalizing coagulation laboratories. Bivalirudin is titrated to achieve an aPTT goal of 60–90 s in patients with standard risk bleeding, and 50–60 s for those at high risk bleeding. Antiplatelet agents are also used, and include Aspirin, with confirmation of therapeutic range by TEG-PM or VerifyNow. Steroids (prednisone) is used for signs of inflammation as in the Stanford protocol.

**Table 1:** Pediatric VAD anticoagulation protocols.

	**Post op**	**Long term**	**Anti-platelets**	**Monitoring**	**Other**
Edmonton protocol (5)	Heparin	Enoxaparin ≤ 12 months Warfarin >12 months	Dual antiplatelet therapy: Dipyridamole 4 mg/kg/day Aspirin 1 mg/kg/day	TEG-PM	
Stanford protocol (49)	Heparin	Enoxaparin ≤ 12 months Warfarin >12 months	Triple anti-platelet therapy: Aspirin 30 mg/ kg/day Dipyridamole 15 mg/kg/day Clopidogrel 1 mg/kg/day	None	Steroids for signs of inflammation
DTI (ACTION) harmonization protocol (10)	Bivalirudin	Bivalirudin transitioned to Couamdin	Aspirin	TEG-PM or VerifyNow	Steroids for signs of inflammation

*TEG-PM, Thromboelastography with Platelet Mapping; ACTION, Advanced Cardiac Therapies Improving Outcomes Network; DTI, Direct Thrombin Inhibitor*.

### Heparin

Unfractionated heparin (UFH) is the first-line anticoagulant therapy in the children with heart disease. It is the most common anticoagulant agent for the immediate post-operative VAD placement period. The criteria to start it are typically absence of bleeding and surgical hemostasis. Heparin dose is typically titrated thereafter using a target activated partial thromboplastin time (aPTT), most commonly between 50 and 80 s (6).

However, heparin has many known challenges but mostly related to the heterogeneous biochemical composition of different molecular weight glycosaminoglycans as well as its dependence upon AT. The heterogeneity in composition of commercial formulations of UFH can result in a wide inter-patient variability in dose–response. The differences in AT levels that change from fetal to adult life is a key additional variant that needs to be considered in infants and children managed with heparin. Heparin-induced thrombocytopenia (HIT) and the osteopenia with its long term use are reported and known risks of heparin exposure (15). There have also been reports of potentially genetic variances in the AT protein with point mutations at the glycosylation sites where AT binds to heparin, which may impair the strength of the covalent bonding (50).

Measuring the heparin response to titrate heparin dose is a known area of controversy. Activated clotting time (ACT) is a point of care assay which measures time to clot initiation of whole blood. This test has several limitations including variability in technical methods for clot activation and detection. Additionally, ACT is not specific to the effects of heparin and reflect other physiologic factors that impact coagulation (51).

Although aPTT is the most commonly used blood test to titrate heparin anticoagulation effect in pediatrics VAD patients, anti-factor Xa activity is increasingly used (6). The anti-factor Xa activity (anti-Xa) assay chromogenically quantifies the heparin–AT complex, and is therefore highly dependent upon serum AT levels; hence anti-Xa activity is frequently low in neonates and infants (52). AT replacement during the perioperative period to augment heparin response is reported in a number of studies. The threshold for replacing AT is variable and ranges <70% in older children and <60% in neonates (6).

## Bivalirudin

Bivalirudin is an intravenous DTI that inhibits both circulating and fibrin-bound thrombin. It is relatively new and semisynthetic drug. It does not bind to plasma proteins and inhibits both free and clot-bound thrombin. It is not dependent on AT and is less immunogenic than heparin. It may also inhibit platelet adhesion. Bivalirudin has a short half-life of 25–35 min secondary to its intravascular proteolytic degradation and minimal renal clearance (~20%). Clinical studies in pediatric showed safety and efficacy of bivalirudin in different settings including procedural anticoagulant, post cardiac surgery ECMO, heart transplant (53, 54). In a single institution experience in 54 children placed on ECLS for a total of 56 runs, Bivalirudin use showed no differences in outcomes compared to heparin, however resulted in longer freedom from first circuit intervention (55). Bivalirudin has also been used safely in pediatric in cases of contraindications to heparin (i.e., HIT) (56) or when unreliable heparin monitoring exists (i.e., severe hemolysis and hyperbilirubinemia) (57). The DTI experience in children is growing. The collective experience of 10 pediatric VAD centers across North America using bivalirudin in children supported with paracorporeal VADs was recently described by VanderPluym et al. Bivalirudin use in this settings was associated with a lower or a comparable rate of stroke to other known anticoagulation and antiplatelet regimens. The risk of bleeding was also comparable in older children supported with intracorporeal continuous flow VADs (10).

The starting dose of bivalirudin is 0.3 mg/kg/h, which is halved in patients with renal dysfunction Table 2. Measuring aPTT is the standard test to monitor anticoagulation with DTI with goal ranging between 2 and 3 times baseline aPTT value for standard bleeding risk patients, and 1.5–2 times baseline aPTT value for high bleeding risk patients. Patients are initially monitored every 4 h until a stable Bivalirudin dosing is achieved, and then daily Table 3. In a small retrospective case-control study for pediatric patients on ECMO, time to reach goal therapeutic anticoagulation level was shorter in bivalirudin compared to heparin (11 vs. 29 h, *P* = 0.01) (58). INR is frequently elevated in patients on Bivalirudin. This is believed to be a false elevation due to the interaction between DTI and the thromboplastin and tissue factor contained in the PT assay, along with the high molar concentrations of the DTIs needed to achieve their anticoagulant effect (59). aPTT sample contamination is infrequently encountered. While hepzyme can be used to neutralize contaminating heparin, concomitant INR is used to identify contamination since INR will not increase with heparin contamination alone. INR monitoring can also be utilized more when bivalirudin dose is escalated which is observed to happen over time (10) at which time there would also be a more “plateau” effect rather than linear aPTT to dose response effect. Some explanation of this phenomenon was related to the increased in fibrinogen levels over time in these patients, which in turn competes with the bivalirudin for the thrombin binding sites (58).

**Table 2:** ACTION initial bivalirudin dosing.

**Goal: aPTT**	
Standard risk (of bleeding): aPTT 60–90 (~2-3x baseline)^*^	
High risk (of bleeding): aPTT 50–60 (~1.5-2x baseline)^**^	
Renal function (GFR)	Initial dosing
Normal (>60 ml/min/1.73 m^2^)	0.3 mg/kg/h IV infusion
Mild-moderate (30–60 ml/min/1.73 m^2^)	0.2 mg/kg/h IV infusion
Severe (<30 ml/min/1.73 m^2^)	0.1 mg/kg/h IV infusion

* Baseline aPTT may be elevated if previously on AC agent, therefore use age/intuitional normative range.

** *Given the risk of early bleeding, most of patients will begin in the “High risk” category for the 1st several days on support, then transition to “Standard risk”*.

**Table 3:** ACTION maintenance bivalirudin titration.

**Goal: aPTT**	
Standard risk (of bleeding): aPTT 60–90 (~2-3x baseline)	
High risk (of bleeding): aPTT 50–60 (~1.5-2x baseline)	
If aPTT 5–15 s out of range:	Increase or decrease by 15% (round up to closest 2nd decimal) Recheck 2–3 h after dose change
If aPTT in target range, no change	Recheck 2–3 h, then can decrease frequency when stable
If aPTT ≥15–30 s out of range	Increase or decrease by 25% (round up to closest 2nd decimal Recheck 2–3 h after dose change
If aPTT >3x baseline or ~120 s	With normal renal function: hold 15 min and reduce by 30% With mild to moderate renal dysfunction: hold for 45 min and reduce by 40% With severe renal dysfunction: hold 2 h and recheck PTT before restarting

The cost effectiveness of bivalirudin remains an area of controversy since the drug by itself is far more expensive than heparin. Ongoing efforts are undergoing to understand this more, however in ECMO patients and when including the cost of AT replacement and the various laboratory monitoring, the overall cost for anticoagulation was decreased in patients receiving bivalirudin, particularly in children younger than 1 year (58).

### Antiplatelet Agents

Antiplatelet therapy has been the main focus for evolving pediatric VAD anticoagulation protocols which could have been driven based on the early Berlin Heart EXCOR experience. Dual and triple antiplatelet therapy had dominated most of the earlier regimens, however with the evolving of improved and consistent anticoagulation agents, i.e., DTI, the emphasis on the dosing with multi antiplatelet agents' nature has been less popular. We use primarily one agent at our institution with consideration to switch or add another agent if there is evidence of sub-therapeutic effect based on the available laboratory tests (Platelets mapping and VerifyNow). TEG-PM is a functional assay to test for pathologic bleeding etiology or to confirm therapeutic anticoagulation and antiplatelet effects. In this testing technology, the thrombus integrity is measured mechanically and in real time. It is then represented by a characteristic curve, which is interpreted based on normal reference. The clot strength is measured by the maximal amplitude (MA) on the curve, which is also used to evaluate ADP (adenosine-diphosphate) or AA (arachiodonic acid) pathways inhibition, which form the platelets mapping test portion (60). VerifyNow is a functional assay point-of-care testing which evaluates platelet aggregation by a turbidimetric-based optical detection (61).

While antiplatelet therapy is historically the main focus for pediatric VAD anticoagulation, and it remains an essential part of it, there is some evidence that TEG-PM in pediatric VAD patients have high intraindividual variability (62). There are also some preliminary reports of a significant role for timing of drug administration and interval frequency which might help improve our understanding of cases of suboptimal response. Adult VAD literature reports a common practice for addition of antiplatelet agents to the anticoagulation regimen, with acknowledgment for treatment resistance and consideration for routine assessment for such.

## Vitamin K Antagonist (VKA)

Most adult VAD patients supported on intracorporeal devices are maintained on vitamin K antagonist (VKA) such as warfarin with goal INR that varies mostly between 2 and 3 (63). Similarly, pediatric VAD patients with intracorporeal devices are primarily maintained on VKA with goal INR ranging from 2 to 3.5, which may vary based on patients and device factors. VKA are typically started when the patient is extubated, with advancing diet. VKA effect is heavily influenced by diet, status of illness, and polypharmacy. Studies to estimate time in therapeutic range for pediatric patients are lacking, but reports from adult studies show the time in therapeutic range as only 30–50% (64).

## DOAC

Direct oral anticoagulants (DOAC) are increasingly used in place of VKA. DOAC medications include factor Xa inhibitors (apixaban and rivaroxaban) and DTI (dabigatran). Few positive features are ease of administration with oral formulations, lack of dependency on AT, lack of dietary interaction, and the requiring less monitoring. Indications for use in the adult populations include venous thromboembolism prophylaxis and atrial fibrillation. A prospective, randomized, open label phase II multi-national clinical trial of a direct oral anticoagulant (DOAC), Apixaban, in children and infants with congenital and acquired heart disease is currently underway (48).

DOAC use in adult VAD patients is not well-established. Two examples of such experience are a small study of seven patients using dabigatran with no excess rate of bleeding or thrombosis (65) and a single center, randomized trial of dabigatran vs. warfarin which was terminated early due to increased thromboembolic events associated with dabigatran (66). Additionally, given the negative experience with dabigatran use in mechanical heart valves, routine use could not be recommended without more reassuring clinical trials (67).

## Pre-OP Management

Prior to VAD device placement, laboratory assessment is recommended to identify potential risk factors for adverse events related to immediate and long-term anticoagulation use. These can include platelet count, prothrombin time and International Normalized Ratio (INR), partial thromboplastin time, fibrinogen, basic metabolic panel (BMP). Other optional labs include Thromboelastography with platelet mapping (TEG with PM), C-reactive protein CRP, Lactate dehydrogenase (LDH), cystatin C, screening for heparin induced thrombocytopenia (HIT screen). In addition to these labs, past medical history is obtained to identify prior thrombotic and bleeding events along with any family history that predisposes the patient to complications.

Thrombophilia evaluation can be completed if there is past or family history of thrombotic events suggesting either an acquired or inherited thrombophilia. These labs include: anticardiolipin, beta-2 glycoprotein, lupus anticoagulant, factor V Leiden, prothrombin gene 20210A mutation, AT3, and proteins C and S.

## Post OP Management

Postoperative management details are critical in establishing a stable and sustained VAD circulation. Cognizant anticoagulation management and monitoring in the early post-operative periods, as well as during switching between agents is highly recommended. The majority of bleeding events and thromboembolic events in pediatric VAD patients occurred while patients were on unfractionated heparin or transitioning between heparin and warfarin (47).

At our institution, postoperative inpatient management include antiplatelet and anticoagulation strategies in the early postoperative period Table 4. This is initiated and managed by the VAD team in consultation with the CICU. Standard heparin anticoagulation for cardiopulmonary bypass is utilized with full protamine reversal in the operating room. Following admission to CICU, once hemostasis is achieved (chest tube output <2 cc/kg/h for 4 consecutive hours) and following correction of acquired coagulopathy (coagulation labs normalizing with platelet count >100,000, INR <1.4, aPTT <40 s and fibrinogen > 100), we start bivalirudin with initial aPTT goal of 50–60, and escalate the goal to 70–90 over the next few days, based on the appearance of the pump. Antiplatelet agents are usually introduced based on appearance of the pump (started sooner if formation of fibrin or clot). We utilize TEG with platelet mapping, and delay initiation of antiplatelet agents until clot strength is >70 mm. As such, antiplatelet agents are generally started 1–2 weeks after VAD implantation for patients on bivalirudin and 5–7 days for patients on warfarin therapy supported on intracorporeal devices. Patient supported on paracorporeal devices remain on bivalirudin for the duration of VAD support, based on data supporting the low rate of hemocompatibility related adverse events using DTI. Patients supported on intracorporeal devices are bridged with bivalirudin or unfractionated heparin to VKA, with target INR goal of 2–3.5. A dedicated Multidisciplinary Anticoagulation Management Team is crucial to optimal anticoagulation management in VAD patients and should include a pharmacist, Cardiac Critical Care, and VAD physicians.

**Table 4:** Inpatient anticoagulation management of pediatric VAD patients.

**Prior to VAD placement**	
Baseline labs	
Minimum	Platelet count, PT, INR, PTT, fibrinogen, basic metabolic panel (BMP)
Additional	TEG with PM, CRP, LDH, cystatin C, HIT screen
Medical and family history	Thrombotic and bleeding events
If + medical or family history	Anticardiolipin, beta-2 glycoprotein, lupus anticoagulant, factor V Leiden, prothrombin gene 20210A mutation, antithrombin 3, and proteins C and S
**Post VAD placement**	
Post-operative day 0	Following hemostasis^a^, Bivalirudin is started at 0.1–0.3 mg/kg/h with initial aPTT goal of 50–60, and escalating the goal to 70–90 over the next few days
Post-Operative day 1 and after	
Paracorporeal	Bivalirudin remains for the duration of VAD support Antiplatelet agents are started within 1–2 weeks, and are delayed until clot strength is >70 mm based on TEG-PM. They may start sooner if formation of fibrin or clot on the pump
Intracorporeal	Bivalirudin or unfractionated heparin is bridged to VKA, with target INR goal of 2–3.5 Antiplatelet agents are started within 5–7 days

a *Hemostasis: chest tube output <2 cc/kg/h for 4 consecutive hours, correction of acquired coagulopathy with platelet count >100,000, INR <1.4, aPTT <40 and fibrinogen > 100*.

### Procedure Anticoagulation Management

Given that the majority of bleeding and thromboembolic events in pediatric VAD patients occur while patients were on unfractionated heparin or transitioning off heparin, it is imperative that the anticoagulation and antithrombosis around any procedure is carefully managed. Antithrombosis considerations surrounding procedures are specific to the clinical state of the patient and the pump, and the bleeding risk of the procedure. As such, decisions to be made in conjunction with all team members, as risk of thrombotic and bleeding events is naturally highest during procedures, even those deemed relatively minor. These decisions need to be individualized taking into consideration the thrombotic risk of the patient and pump, weighed against the bleeding risk of the procedures. Nonessential procedures should be deferred till after VAD support, and essential procedures require discussion with all team members to understand bridging plan so that cessation of antithrombotic agents is minimized, while procedural hemostasis is maximized.

As a general principle we recommend the following:

- For procedures deemed low risk of bleeding

Continue all antiplatelet agents, and dose reduce anticoagulation agent for lower therapeutic target around the procedures

- For procedures deemed high risk of bleeding

* Hold antiplatelet agents around 3 days prior to procedure
* Hold bivalirudin ~ 1–4 h prior to skin incision (depending on renal function)
* Hold unfractionated heparin 4 h prior to skin incision
* VKA, start holding 3 days prior to procedure with daily INR measurement. Once INR <2, then start LMWH or continuous infusion of DTI or UFH
* LMWH, hold night before and morning of procedure. Consider bridging with UFH or DTI depending on thrombotic risk of pump and patient.

Prompt re-initiation of anticoagulation is required once hemostasis achieved and in discussion with all team members to ensure risk of re-bleeding is minimized, with hourly pump examinations for paracorporeal pumps for 24 h post procedure.

## Hemolysis

Hemolysis is a common phenomenon seen after VAD implantation, particularly in children. Patient size and anatomy, Device hemocompatibility profile, and shear stress, as well as anticoagulant management all contribute to increased hemolysis. Hemolysis can also be an important marker of device thrombosis, and thus early recognition is important. Routine laboratory monitoring with plasma free hemoglobin (product of erythrocyte destruction), lactate dehydrogenase (LDH), or haptoglobin should be considered. Increasing plasma free hemoglobin is associated with renal dysfunction. Supplemental iron, folic acid, and erythropoietin, along with a lower threshold for transfusions were recommended by (11).

### VAD Acquired Hemostatic Pathophysiologies

High shear stress effects result in loss of high molecular weight von Willebrand (vWF) multimers which significantly reduces its size which in turn provides less hemostasis. Acquired von Willebrand syndrome (AVWS) is reported in patients following VAD placement. In a prospective cohort for 37 adult patients, significant loss of HMW vWF multimers was reported within 30 days of CF-VAD implantation, yet only 10 of the 37 patients experienced bleeding complications. This suggested that loss of HMW vWF multimers alone could not predict bleeding risk (68). Device hemocompatiblity is likely to be a factor in this syndrome, as AVWS in patients with HM III was less severe than in patients with HMII, which also correlated with less bleeding symptoms (69).

Platelets aggregation function is also believed to be impaired in VAD patients. In adult VAD patients, and unlike AVWS, platelet function defects were found equally present with both VAD types, HM II, and HM III (69).

## Future Directions

Ventricular assist devices are increasingly used in children, not only as a bridge to transplantation, but also as bridge to decision and many patients are starting to discharge to home. Safe and effective anticoagulation is required for successful management of children supported with ventricular assist devices. Developmental hemostasis, device hemocompatibility, plastic to body ratio, surgical variable techniques, lack of knowledge on pharmacokinetics of anticoagulants, and wide variability in anticoagulation protocols have all contributed to increased incidence of bleeding and thromboembolic complications. New collaborative learning networks, such as the ACTION network, provide opportunities to define best practices, optimize and reduce anticoagulation related adverse events. Changes in anticoagulation strategy from the use of heparin to a DTI such as bivalirudin have helped reduce bleeding and clotting complications. The future of this field lies in the development of VAD with improved biocompatibility profiles that can help reduce the need for anticoagulants therapies and reduce the risk of bleeding and thrombosis complications.

## Author Contributions

The main content of the article was provided by MG. CV and RT reviewed, minimally edited, and confirmed the article materials provided. All authors contributed to the article and approved the submitted version.

## Conflict of Interest

The authors declare that the research was conducted in the absence of any commercial or financial relationships that could be construed as a potential conflict of interest.

## Abbreviations

ACTION: Advanced Cardiac Therapies Improving Outcomes Network
AT: Antithrombin 3
BMP: basic metabolic panel
CRP: C-Reactive Protein
DTI: Direct Thrombin Inhibitor
HIT: heparin-induced thrombocytopenia
INR: international normalized ratio
LDH: Lactate dehydrogenase
PT: prothrombin time
PTT: Partial thromboplastin time
TEG-PM: Thromboelastography with Platelet Mapping
VAD: Ventricular assist devices.

## References

[1] Fraser CD Jr Jaquiss RD Rosenthal DN Humpl T Canter CE Blackstone EH. Prospective trial of a pediatric ventricular assist device. N Engl J Med. (2012) 367:532–41. 10.1056/NEJMoa1014164 22873533

[2] Zafar F Castleberry C Khan MS Mehta V Bryant R III Lorts A. Pediatric heart transplant waiting list mortality in the era of ventricular assist devices. J Heart Lung Transplant. (2015) 34:82–88. 10.1016/j.healun.2014.09.018 25447574

[3] Morales DLS Rossano JW VanderPluym C Lorts A Cantor R St Louis JD. Third annual pediatric interagency registry for mechanical circulatory support (pedimacs) report: preimplant characteristics and outcomes. Ann Thorac Surg. (2019) 107:993–1004. 10.1016/j.athoracsur.2019.01.038 30817920

[4] Thangappan K Zafar F Morales D. Milestone: more than 1,200 children bridged to transplantation with MCS. In: *AATS Confernce Presentation*. American Association for Thoracic Surgery (2020).

[5] Steiner ME Bomgaars LR Massicotte MP. Berlin heart EXCOR pediatric VAD IDE study investigators antithrombotic therapy in a prospective trial of a pediatric ventricular assist device. ASAIO J. (2016) 62:719–27. 10.1097/MAT.0000000000000434 27556152 PMC5098459

[6] Huang JY Monagle P Massicotte MP VanderPluym CJ. Antithrombotic therapies in children on durable ventricular assist devices: a literature review. Thromb Res. (2018) 172:194–203. 10.1016/j.thromres.2018.02.145 29501323

[7] Attard C van der Straaten T Karlaftis V Monagle P Ignjatovic V. Developmental hemostasis: age-specific differences in the levels of hemostatic proteins. J Thromb Haemost. (2013) 11:1850–4. 10.1111/jth.12372 23927605

[8] Byrnes JW Bhutta AT Rettiganti MR Gomez A Garcia X Dyamenahalli U. Steroid therapy attenuates acute phase reactant response among children on ventricular assist device support. Ann Thorac Surg. (2015) 99:1392–8. 10.1016/j.athoracsur.2014.11.046 25669667

[9] Byrnes JW Prodhan P Williams BA Schmitz ML Moss MM Dyamenahalli U. Incremental reduction in the incidence of stroke in children supported with the Berlin EXCOR ventricular assist device. Ann Thorac Surg. (2013) 96:1727–33. 10.1016/j.athoracsur.2013.06.012 23998407

[10] VanderPluym CJ Cantor RS Machado D Boyle G May L Griffiths E. Utilization and outcomes of children treated with direct thrombin inhibitors on paracorporeal ventricular assist device support. ASAIO. (2020) 66:939–45. 10.1097/MAT.0000000000001093 32740356

[11] Tume SC Conway J Ryan KR Philip J Fortkiewicz JM Murray J. Developments in pediatric ventricular assist device support. World J Pediatr Congenit Heart Surg. (2019) 10:759–68. 10.1177/2150135119880890 31663841

[12] Miera O Kirk R Buchholz H Schmitt KR VanderPluym C Rebeyka IM. A multicenter study of the HeartWare ventricular assist device in small children. J Heart Lung Transplant. (2016) 35:679–81. 10.1016/j.healun.2016.01.019 26922273

[13] Lorts A Eghtesady P Mehegan M Adachi I Villa C Davies R. Outcomes of children supported with devices labeled as temporary or short term: a report from the pediatric interagency registry for mechanical circulatory support. J Heart Lung Transplant. (2018) 37:54–60. 10.1016/j.healun.2017.10.023 29174220

[14] Kulat B Russell HM Sarwark AE Zingle R Moss ST Mongé MC. Modified tandem heart ventricular assist device for infant and pediatric circulatory support. Ann Thorac Surg. (2014) 93:1437–41. 10.1016/j.athoracsur.2014.05.093 25282205

[15] VanderPluym CJ Adachi I Niebler R Griffiths E Fynn-Thompson F Chen S. Outcomes of children supported with an intracorporeal continuous-flow left ventricular assist system. J Heart Lung Transplant. (2019) 38:385–93. 10.1016/j.healun.2018.09.015 30391197

[16] O'Connor MJ Lorts A Davies RR Fynn-Thompson F Joong A Maeda K. Early experience with the HeartMate 3 continuous-flow ventricular assist device in pediatric patients and patients with congenital heart disease: a multicenter registry analysis. J Heart Lung Transplant. (2020) 39:573–9. 10.1016/j.healun.2020.02.007 32111350

[17] Baldwin JT Adachi I Teal J Almond CA Jaquiss RD Massicotte MP. Closing in on the PumpKIN trial of the Jarvik 2015 ventricular assist device. Semin Thorac Cardiovasc Surg Pediatr Card Surg Annu. (2017) 20:9–15. 10.1053/j.pcsu.2016.09.003 28007073 PMC5189910

[18] Parekh D Jeewa A Tume SC Dreyer WJ Pignatelli R Horne D. Percutaneous mechanical circulatory support using impella devices for decompensated cardiogenic shock: a pediatric heart center experience. ASAIO J. (2018) 64:98–104. 10.1097/MAT.0000000000000581 28394814

[19] Batsides G Massaro J Cheung A Soltesz E Ramzy D Anderson MB. Outcomes of impella 5.0 in cardiogenic shock: a systematic review and meta-analysis. Innovations. (2018) 13:254–60. 10.1097/IMI.0000000000000535 30142110

[20] Monagle P Barnes C Ignjatovic V Furmedge J Newall F Chan A. Developmental haemostasis. Impact for clinical haemostasis laboratories. Thromb Haemost. (2006) 95:362–72. 10.1160/TH05-01-0047 16493500

[21] Kuhle S Male C Mitchell L. Developmental hemostasis: pro- and anticoagulant systems during childhood. Semin Thromb Hemost. (2003) 29:329–38. 10.1055/s-2003-42584 14517745

[22] Andrew M Paes B Milner R Johnston M Mitchell L Tollefsen DM. Development of the human coagulation system in the full-term infant. Blood. (1987) 70:165–172. 10.1182/blood.V70.1.165.165 3593964

[23] Andrew M Vegh P Johnston M Bowker J Ofosu F Mitchell L. Maturation of the hemostatic system during childhood. Blood. (1992) 80:81998. 10.1182/blood.V80.8.1998.bloodjournal8081998 1391957

[24] Ignjatovic V Summerhayes R Than J Gan A Monagle P. Therapeutic range for unfractionated heparin therapy: age-related differences in response in children. J Thromb Haemost. (2006) 4:2280–2. 10.1111/j.1538-7836.2006.02136.x 16856975

[25] Payne RM Burns KM Glatz AC Li D Li X Monagle P. Pediatric heart network investigators. A multi-national trial of a direct oral anticoagulant in children with cardiac disease: design and rationale of the Safety of ApiXaban On Pediatric Heart disease On the preventioN of Embolism (SAXOPHONE) study. Am Heart J. (2019) 217:52–63. 10.1016/j.ahj.2019.08.002 31493728 PMC6861679

[26] Michelson AD. Platelet function in the newborn. Semin Thromb Hemost. (1998) 24:507–12. 10.1055/s-2007-996049 10066145

[27] Manlhiot C Menjak IB Brandão LR Gruenwald CE Schwartz SM Sivarajan VB. Risk, clinical features, and outcomes of thrombosis associated with pediatric cardiac surgery. Circulation. (2011) 124:1511–9. 10.1161/CIRCULATIONAHA.110.006304 21911785

[28] Odegard KC Zurakowski D Hornykewycz S DiNardo JA Castro RA Neufeld EJ. Evaluation of the coagulation system in children with two-ventricle congenital heart disease. Ann Thorac Surg. (2007) 83:1797–803. 10.1016/j.athoracsur.2006.12.030 17462402

[29] Odegard KC McGowan FX Jr Zurakowski D Dinardo JA Castro RA del Nido PJ. Procoagulant and anticoagulant factor abnormalities following the Fontan procedure: increased factor VIII may predispose to thrombosis. J Thorac Cardiovasc Surg. (2003) 125:1260–7. 10.1016/S0022-5223(02)73605-2 12830042

[30] Jaquiss RDB Humpl T Canter CE Morales DLS Rosenthal DN Fraser CD. Postapproval outcomes: the berlin heart EXCOR pediatric in North America. ASAIO. (2017) 63:193–197. 10.1097/MAT.0000000000000454 28234657

[31] Connors JM. Anticoagulation management of left ventricular assist devices. Am J Hematol. (2015) 90:175–8. 10.1002/ajh.23836 25163820

[32] Kirklin JK Naftel DC Pagani FD Kormos RL Stevenson LW Blume ED. Seventh INTERMACS annual report: 15,000 patients and counting. J Heart Lung Transplant. (2015) 34:1495–504. 10.1016/j.healun.2015.10.003 26520247

[33] Colombo PC Mehra MR Goldstein DJ Estep JD Salerno C Jorde UP. Comprehensive analysis of stroke in the long-term cohort of the MOMENTUM 3 study. Circulation. (2019) 139:155–68. 10.1161/CIRCULATIONAHA.118.037231 30586698

[34] Bartoli CR Ailawadi G Kern JA. Diagnosis, nonsurgical management, and prevention of LVAD thrombosis. J Card Surg. (2014) 29:83–94. 10.1111/jocs.12238 24267706

[35] Dang G Epperla N Muppidi V Sahr N Pan A Simpson P. Medical management of pump-related thrombosis in patients with continuous-flow left ventricular assist devices: a systematic review and meta-analysis. ASAIO J. (2017) 63:373–85. 10.1097/MAT.0000000000000497 27984314 PMC5468512

[36] Mehra MR Naka Y Uriel N Goldstein DJ Cleveland JC Jr Colombo PC. A fully magnetically levitated circulatory pump for advanced heart failure. N Engl J Med. (2017) 376:440–50. 10.1056/NEJMoa1610426 27959709

[37] Barac YD Wojnarski CM Junpaparp P Patel CB Schroder JN Milano CA. Early outcomes with durable left ventricular assist device replacement using the HeartMate 3. J Thorac Cardiovasc Surg. (2020) 160:132–9. 10.1016/j.jtcvs.2019.09.151 31740114 PMC7325332

[38] Barac YD Schroder JN Daneshmand MA Patel CB Milano CA. Heartmate III Replacement for Recurring Left Ventricular Assist Device Pump Thrombosis. ASAIO, J., (2018) 424–6. 10.1097/MAT.0000000000000713 29112021

[39] Hetzer R Loebe M Potapov EV Weng Y Stiller B Hennig E. Circulatory support with pneumatic paracorporeal ventricular assist device in infants and children. Ann Thorac Surg. (1998) 66:1498–506. 10.1016/S0003-4975(98)00914-X 9875742

[40] Rimsans J Sylvester KW Connors JM. Direct Thrombin Inhibitor for LVAD Thrombosis: A Closer Look. Clin Appl Thromb Hemost., (2017) 405–9. 10.1177/1076029616672583 27765812

[41] Weeks P Sieg A Rajapreyar I Nathan S Jumean M Patel M. Bivalirudin for left ventricular assist device thrombosis. J Thromb Thrombolysis. (2018) 46:496–50. 10.1007/s11239-018-1725-z 30120650

[42] Kiernan MS Katz JN. Operating in the dark. When is surgery necessary for left ventricular assist device hemolysis. Circ Heart Fail. (2016) 9:3141. 10.1161/CIRCHEARTFAILURE.116.003141 27166249

[43] Chetan D Buchholz H Bauman M Anand V Holinski P Conway J. Successful treatment of pediatric ventricular assist device thrombosis. ASAIO J. (2018) 64:28–32. 10.1097/MAT.0000000000000606 28604570

[44] Lorts A Smyth L Gajarski RJ VanderPluym CJ Mehegan M Villa CR. The creation of a pediatric heart care learning network: the ACTION Quality Improvement Collaborative. ASAOP J. (2020) 66:441–6. 10.1097/MAT.0000000000001133 32224822

[45] Levesque AA Lewin AR Rimsans J Sylvester KW Coakley L Melanson F. Development of multidisciplinary anticoagulation management guidelines for patients receiving durable mechanical circulatory support. Clin Appl Thromb Hemost. (2019) 25:1076029619837362. 10.1177/1076029619837362 30907120 PMC6714942

[46] Kirklin JK Pagani FD Kormos RL Stevenson LW Blume ED Myers SL. Eighth annual INTERMACS report: special focus on framing the impact of adverse events. J Heart Lung Transplant. (2017) 36:1080–6. 10.1016/j.healun.2017.07.005 28942782

[47] Huang JY Ignjatovic V Sheridan BJ Mathew J D'Udekem Y Brink J. Bleeding and thrombotic events occur early in children on durable ventricular assist devices. Thromb Res. (2019) 173:65–70. 10.1016/j.thromres.2018.11.019 30476715

[48] Rosenthal DN Lancaster CA McElhinney DB Chen S Stein M Lin A. Impact of a modified anti-thrombotic guideline on stroke in children supported with a pediatric ventricular assist device. J Heart Lung Transplant. (2017) 1250–7. 10.1016/j.healun.2017.05.020 28606584

[49] Sylvia LM Ordway L Pham DT DeNofrio D Kiernan M. Bivalirudin for treatment of LVAD thrombosis: a case series. ASAIO J. (2014) 60:744–7. 10.1097/MAT.0000000000000122 25072553

[50] Fischer R Sachs UJ Heidinger KS Eisenburger D Kemkes-Matthes B. Prevalence of hereditary antithrombin mutations is higher than estimated in patients with thrombotic events. Blood Coagul Fibrinolysis. (2013) 24:444–8. 10.1097/MBC.0b013e32835cc143 23429250

[51] Baumann Kreuziger L Karkouti K Tweddell J Massicotte MP. Antithrombotic therapy management of adult and pediatric cardiac surgery patients. J Thromb Haemost. (2018) 16:2133–46. 10.1111/jth.14276 30153372

[52] Nair AG Oladunjoye OO Trenor CC III LaRonde M van den Bosch SJ Sleeper LA. An anticoagulation protocol for use after congenital cardiac surgery. J Thorac Cardiovasc Surg. (2018) 156:343–52.e4. 10.1016/j.jtcvs.2018.02.106 29706371

[53] Hasija S Talwar S Makhija N Chauhan S Malhotra P Chowdhury UK. Randomized controlled trial of heparin versus bivalirudin anticoagulation in acyanotic children undergoing open heart surgery. J Cardiothorac Vasc Anesth. (2018) 32:2633–40. 10.1053/j.jvca.2018.04.028 30482701

[54] Zaleski KL DiNardo JA Nasr VG. Bivalirudin for pediatric procedural anticoagulation: a narrative review. Anesth Analg. (2019) 128:43–55. 10.1213/ANE.0000000000002835 29461391

[55] Schill MR Douds MT Burns EL Lahart MA Said AS Abarbanell AM. Is anticoagulation with bivalirudin comparable to heparin for pediatric extracorporeal life support? results from a high-volume center. Artif Organs. 45:15–21. 10.1111/aor.13758 32557733

[56] Yuan SM. Heparin-induced thrombocytopenia in pediatrics following cardiopulmonary bypass. J Coll Physicians Surg Pak. (2019) 10:986–92. 10.29271/jcpsp.2019.10.986 31564275

[57] Ezetendu C Jarden A Hamzah M Stewart R. Bivalirudin anticoagulation for an infant with hyperbilirubinemia and elevated plasma-free hemoglobin on ECMO. J Extra Corpor Technol. (2019) 51:26–8. 30936585 PMC6436166

[58] Hamzah M Jarden AM Ezetendu C Stewart R. Evaluation of bivalirudin as an alternative to heparin for systemic anticoagulation in pediatric extracorporeal membrane oxygenation. Pediatr Crit Care Med. (2020) 21:827–34. 10.1097/PCC.0000000000002384 32404633

[59] Hohlfelder B DeiCicchi D Sylvester KW Connors JM. Development of a predictive nomogram for the change in PT/INR upon discontinuation of bivalirudin as a bridge to warfarin. Clin Appl Thromb Hemost. (2017) 23:487–93. 10.1177/1076029616638505 26994297

[60] Corliss BM Freedman R Brennan MM Smith J Nerva JD Harris NS. Laboratory assessments of therapeutic platelet inhibition in endovascular neurosurgery: complication prediction using the Verify Now P2Y12 assay and thromboelastography with platelet mapping. J Neurosurg. (2020) 1–9. 10.3171/2019.12.JNS192396 32084635

[61] Smith JW Steinhubl SR Lincoff AM Coleman JC Lee TT Hillman RS. Rapid platelet-function assay: an automated and quantitative cartridge-based method. Circulation. (1999) 99:620–5. 10.1161/01.CIR.99.5.620 9950658

[62] Ferguson LP Duong P Pearce KF Murphy P Biss TT. Monitoring of antiplatelet therapy in children on ventricular assist device support: comparison of multiplate and thromboelastography platelet mapping. ASAIO J. (2019) 65:84–93. 10.1097/MAT.0000000000000768 29489462

[63] Baumann Kreuziger LM Kim B Wieselthaler GM. Antithrombotic therapy for left ventricular assist devices in adults: a systematic review. J Thromb Haemost. (2015) 13:946–55. 10.1111/jth.12948 25845489

[64] Baumann Kreuziger LM. Management of anticoagulation and antiplatelet therapy in patients with left ventricular assist devices. J Thromb Thrombolysis. (2015) 2015:337–44. 10.1007/s11239-014-1162-6 25549823

[65] Terrovitis JV Ntalianis A Kapelios CJ Vakrou S Diakos N Katsaros L. Dabigatran etexilate as second-line therapy in patients with a left ventricular assist device. Hellenic J Cardiol. (2015) 56:20–5. 25701968

[66] Andreas M Moayedifar R Wieselthaler G Wolzt M Riebandt J Haberl T. Increased thromboembolic events with dabigatran compared with vitamin K antagonism in left ventricular assist device patients: a randomized controlled pilot trial. Circ Heart Fail. (2017) 10:3709. 10.1161/CIRCHEARTFAILURE.116.003709 28500254 PMC5434960

[67] Hilal T Mudd J DeLoughery TG. Hemostatic complications associated with ventricular assist devices. Res Pract Thromb Haemost. (2019) 3:589–98. 10.1002/rth2.12226 31624778 PMC6781923

[68] Crow S Chen D Milano C Thomas W Joyce L Piacentino V 3rd Sharma R. Acquired von Willebrand syndrome in continuous-flow ventricular assist device recipients. Ann Thorac Surg. (2010) 90:1263–9. 10.1016/j.athoracsur.2010.04.099 20868825

[69] Geisen U Brehm K Trummer G Berchtold-Herz M Heilmann C Beyersdorf F. Platelet secretion defects and acquired von Willebrand syndrome in patients with ventricular assist devices. J Am Heart Assoc. (2018) 7:6519. 10.1161/JAHA.117.006519 29331958 PMC5850142

